# Preparation, characterization and cell labelling of strong pH-controlled bicolor fluorescence carbonized polymer dots†

**DOI:** 10.1039/d1ra08092j

**Published:** 2022-01-05

**Authors:** Zengchen Liu, Like Wang, Baodui Wang, Yahong Chen, Fengshou Tian, Yingying Xue, Yanxia Li, Wenping Zhu, Weijie Yang

**Affiliations:** College of Chemistry and Chemical Eningeering, Henan Key Laboratory of Rare Earth Functional Materials, International Joint Research Laboratory for Biomedical Nanomaterials of Henan, Zhoukou Normal University Zhoukou 466001 P. R. China liuzengchen@zknu.cn chen-yh75@163.com; State Key Laboratory of Applied Organic Chemistry, Key Laboratory of Nonferrous Metal Chemistry and Resources Utilization of Gansu Province, Lanzhou University Lanzhou 730000 Gansu P. R. China wangbd@lzu.cn

## Abstract

As a class of important carbon nanomaterial, carbonized polymer dots (CPDs), also called carbon dots (CDs), have aroused wide interest owing to their unique water solubility, fluorescence properties, and rich surface functional groups. However, the directional tuning of the fluorescence properties of CPDs remains incomplete because of the influence of many factors like diameter, solvent and surface groups. Particularly, most carbonized polymer dots are synthesized in a neutral pH environment. Herein, by modulating the pH (strongly acidic or alkaline) of dextrin water solution, bicolor fluorescence emission (blue and yellow) CPDs were prepared by a hydrothermal reaction. Through systematic characterization, it was found that the different fluorescence properties are regulated by the diameters and surface groups of the carbon cores. Simultaneously, the pH value affected the nucleation process. Based on the excellent fluorescence properties, cell fluorescence imaging and cytotoxicity were tested. The bicolor fluorescence CPDs obtained by tuning the pH provide an important theoretical basis for the design of broadband CPDs.

## Abstract 2

Multicolor fluorescence N-doped CPDs from dextrin water solution in strong acidic and alkaline environments were synthesized and characterized, which revealed that pH value plays a vital role in the process of CPD growth.

## Graphical abstract

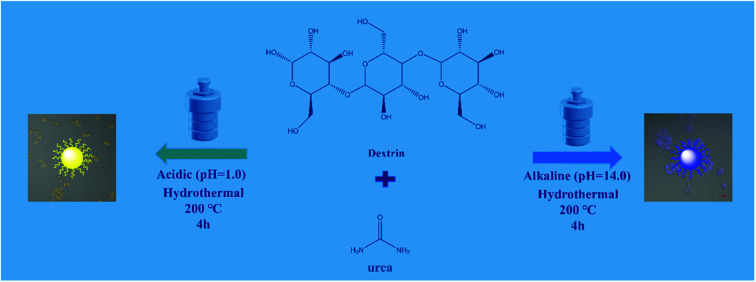

## Introduction

As a kind of promising zero dimensional carbon material, ever since they were discovered,^1^ carbon dots (<10 nm, also called carbonized polymer dots, carbon nanodots and polymeric dots) have received significant attention because they exhibit some prominent features like fluorescence properties, biocompatibility and catalytic properties.^2–6^ To date, enormous progress on fluorescence (from blue to red, even to near infrared) has been made in the field of carbon dots.^7–10^ In particular, the strong multicolor fluorescence properties of carbon dots have been attracting the attention of researchers.^11,12^ This is because multicolor fluorescence carbon dots have attractive application prospects in cell fluorescence bioimaging, which can track cellular changes (direct correlation with diseases) in organisms.^13–15^ Wang *et al.* reported and prepared tricolor CDs from isomers,^16^ and proved that high nitrogen doping, bigger particle sizes and narrower band gaps induced the red shift of fluorescence emission from CDs. Zhang *et al.*^17^ synthesized full-color carbon dots based on a doping strategy and the possible ET mechanisms were proposed. However, the synthesis of multicolor fluorescence CDs depends on organic precursors (aromatic molecules and solvents),^18^ which exhibit potential toxicity and are unsuitable for biological application. Lu *et al.*^19^ prepared multicolor CDs through the hydrothermal process of aromatic glucose under acidic conditions, which was used to construct LEDs of varying colors. In addition, owing to the diversity of raw materials, the fluorescence can be tuned by a lot of internal and external factors. In accordance with previous literature, among the aforementioned conditions, solvent, pH, surface groups and particle size are important factors.^20^ These features lead to the high complexity of carbon dot structures. Among these factors, pH plays an important role in the process of carbon dot growth. However, the effect of the pH value of the polymeric precursor solution on the particle size (carbon core composed of sp^2^ and sp^3^ carbon atoms) and fluorescence of the carbon dots is not yet well understood. Additionally, the relationship of pH, the nanoscale structures (surface groups) and the fluorescence of carbon dots is not clear due to a lack of sufficient theoretical support for elucidating the underlying mechanism.

In this study, we synthesize bicolor fluorescence N-doped CPDs‡‡CPDs represents the abbreviation of carbonized polymer dots. (CPDs-1 and CPDs-2) by controlling the pH value (strong acidic and alkaline environments) of a dextrin water solution. Dextrin comes from the hydrolysis of starch, which is nontoxic and easily available. By the hydrothermal method, blue and yellow fluorescence carbonized polymer dots (CPDs) are obtained. Depending on the strong acidic or alkaline environment, the diameters of the synthesized CPDs are different. In addition, larger particle showed long wavelength emission (strong acidic conditions), which reveals that the pH value plays a vital role in the process of CPD growth. In addition, the IR spectra and UV-vis spectra also exhibited significant difference, indicating that the pH value can affect the surface groups of the CPDs. To reveal the bicolor fluorescence properties of the CPDs, cell fluorescence imaging was achieved, which exhibited excellent channels (blue and yellow) inside the cell. Further, by DFT calculation, the particle size and surface groups synergistically determined the fluorescence activities of the CPDs. Subsequently, we concluded that pH value regulates the particle and surface groups of the CPDs, which can cause bicolor fluorescence emission of the CPDs. The work provides an important paradigm for the pH tuning of water-soluble bicolor CPDs and demonstrates the crucial role of the pH environment in the process of preparing bicolor carbon dots.

## Materials and methods

Hydrochloric acid, sodium hydrate, dextrin (99%, C_6*n*_H_10*n*_O_5*n*_·*x*H_2_O) and urea were purchased from Aladdin Reagent Co. Ltd (Shanghai, China). All the chemicals were used without further purification.

The UV-vis spectra were recorded on a PerkinElmer Lambda-35 UV-vis spectrophotometer. The IR spectra were obtained from a NICOLET 5700 FTIR spectrometer. Fluorescence spectra were obtained on a Cary Eclipse spectrophotometer at room temperature. The fluorescence images of HeLa cells were studied using an Olympus fv1300 laser confocal fluorescence microscope. The SEM images were obtained by F250 thermal field emission scanning electron microscopy. XPS was performed using a Thermo ESCALAB 250XI. The HRTEM spectra were recorded by a Tecnai G2 F20. The AFM data were obtained from a Bruker Dimension Icon. NMR data were obtained a from Bruker AV500 MHz spectrometer.

HeLa cells were cultured in a flask in Dulbecco's modified Eagle's medium supplemented with heat-inactivated bovine serum (10%), 100 U mL^−1^ penicillin and 100 U mL^−1^ streptomycin and were maintained at 37 °C in a humidified atmosphere (5% CO_2_ and 95% air). The cells were seeded in six orifice plates and allowed to adhere for 12 h before treatment. Then, the cells were incubated with fresh media containing 100 μL CPDs-1 and CPDs-2 solutions (10 mg mL^−1^). After 2.0 h, the growth medium was removed, and the cells were washed with saline solution several times. The cover glass was then mounted on a microscope glass slide and studied under a microscope. Fluorescence imaging was performed using an Olympus fv1300 laser confocal fluorescence microscope. The excitation wavelength was 405 nm and the emission collection was in the range of 450–650 nm.

**Scheme 1:**
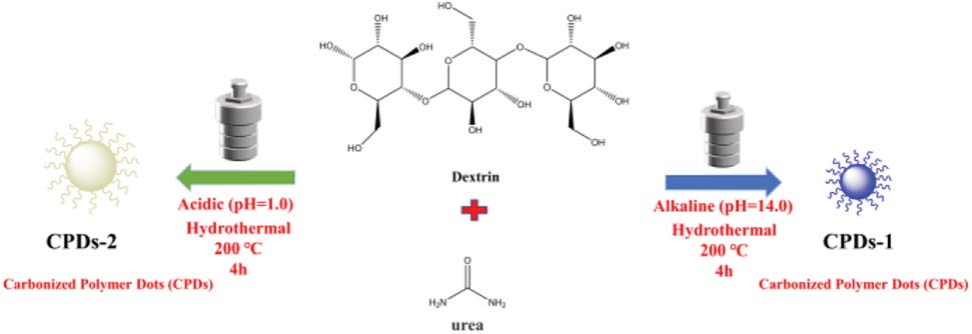
The preparation process of CPDs-1 and CPDs-2.

The synthesis of the nitrogen-doped carbonized polymer dots (CPDs-1 and CPDs-2) was performed *via* a hydrothermal approach. The preparation process of CPDs-1 and CPDs-2 is shown in Scheme 1. Additionally, CPDs-1 and CPDs-2 were obtained under a strong acidic environment (pH = 1) and strong alkaline environment (pH = 14), respectively. Dextrin (2 g) and urea (0.2 g) were dissolved in 50 mL distilled water, and the pH values were tuned to be strongly acidic (pH = 1) and strongly alkaline (pH = 14), respectively. The two solutions exhibited significant difference. Compared to the cloudy solution in the strong acidic environment, the water solution of dextrin and urea was clear in the strong alkaline environment (Fig. S1†). Then, the two solutions were heated to 200 °C and maintained for 4 hours, after which they were cooled naturally to room temperature (25 °C, 12 h after reaction). Subsequently, the mixtures were centrifuged at 8500 rpm for 20 minutes. The supernatant was collected and filtered three times. Then, the two solution were left for 12 hours. The solutions of the carbonized polymer dots (CPDs-1 and CPDs-2) exhibited significant differences (brown and black) under the same reaction conditions except for the pH (acidic and alkaline). Preliminarily, we concluded that the synthesized carbonized polymer dots (CPDs) can represent different forms of existence under different pH conditions. Finally, the water solutions were freeze-dried and the carbonized polymer dots (CPDs-1 and CPDs-2) were obtained. They were characterized by SEM, TEM, XPS, *etc.* In addition, CPDs from an extensive range of pH values (pH = 1.0–14.0) were also obtained to observe some contrasts (Fig. S2†), which showed intense fluorescence emission in strong pH environments.

## Results and discussion

### Fluorescence emission activities of the carbonized polymer dots (CPDs-1 and CPDs-2)

**Fig. 1:**
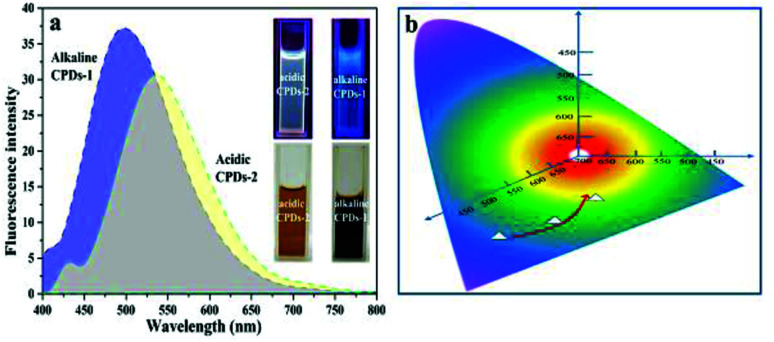
(a) The fluorescence emission spectra (Ex = 405 nm), fluorescence imaging under UV light (Ex = 365 nm,) and apparent performance of the CPDs-1 and CPDs-2 water solutions. (b) The gradually varying fluorescence palette.

By tuning the pH value of the dextrin water solution (strong acidic condition and strong alkaline condition), carbonized polymer dots (CPDs-1 and CPDs-2) were produced by a hydrothermal reaction. Compared with the solution (brownish black) in the alkaline environment, the color (pale brown) of CPDs-2 was lighter, which demonstrated that the carbonation process of dextrin in strongly acidic and alkaline conditions showed some diversity (Fig. 1a). Depending on the natural statuses, the CPDs-1 and CPDs-2 water solutions under UV light showed two different colors of fluorescence emission (blue and yellow). Upon excitation at 405 nm, the fluorescence emission wavelengths of the CPDs-1 and CPDs-2 water solutions were centered at 480 nm and 550 nm (shift of emission >70 nm), respectively. Fig. 1b illustrates the bicolor fluorescence emission positions of the CPDs. As reported, reaction temperature and time obviously affected the fluorescence of the carbonized polymer dots. In this study, pH value also yielded obvious effects on the fluorescence emission characteristics of the carbonized polymer dots. In the following sections, we will discuss the microstructure, spectral characterization, and fluorescence properties of CPDs-1 and CPDs-2.

### SEM and HRTEM characterization of the carbonized polymer dots (CPDs-1 and CPDs-2)

**Fig. 2:**
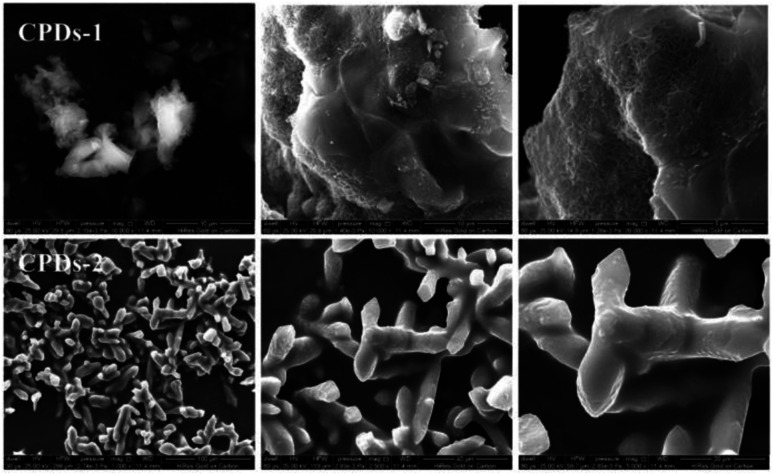
The solid state SEM images of CPDs-1 and CPDs-2.

To investigate the macrostructure of CPDs-1 and CPDs-2 in different pH conditions, scanning electron microscopy (SEM) and high-resolution transmission electron microscopy (HRTEM) were performed and discussed. As shown in Fig. 2, some important differences were observed in the solid state CPDs. Under an alkaline environment, CPDs-1 exhibited honeycomb block construction, while under an acidic environment, CPDs-2 exhibited dendritic construction, which proved that the interaction tractive forces of CPDs-1 and CPDs-2 were different. Additionally, the abundant surface group interaction may cause the aggregation of the carbonized polymer dots.

**Fig. 3:**
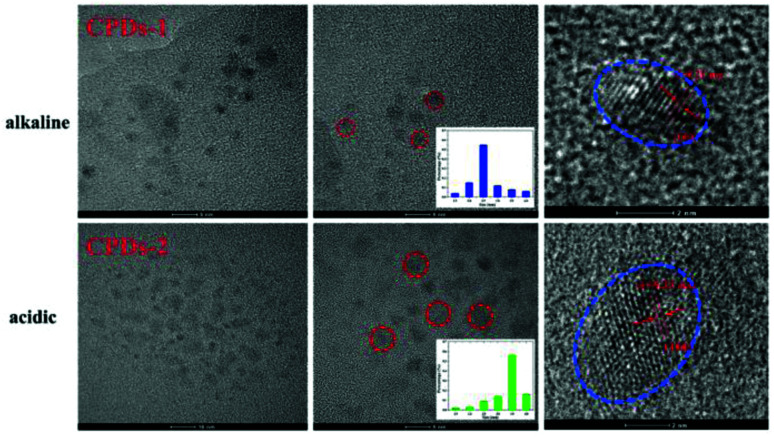
The HRTEM images containing the lattice fringe and size distribution of CPDs-1 and CPDs-2.

Fig. 3 shows the HRTEM images of CPDs-1 and CPDs-2, illustrating their spherical morphology with their average diameters of 2.5 nm ± 0.21 nm and 3.5 nm ± 0.22 nm, respectively. Furthermore, CPDs-1 and CPDs-2 exhibited no obvious morphology differences. Thus, the fluorescence emission variation might be related to the particle size and surface groups of CPDs-1 and CPDs-2. Particularly, bigger particle size can lead to the energy level difference between S0 (ground state) and S1 (excited state), which results in a fluorescence emission change. However, this hypothesis needed to be demonstrated by DFT calculations. In addition, the lattice fringe of CPDs-1 and CPDs-2 was very clear, which can be attributed to the *d* spacing of the graphene plane (100). Furthermore, the same crystal lattices (0.21 nm) indicated that the internal structures of CPDs-1 and CPDs-2 have no significant difference.

### Atomic force microscopy (AFM) characterization

**Fig. 4:**
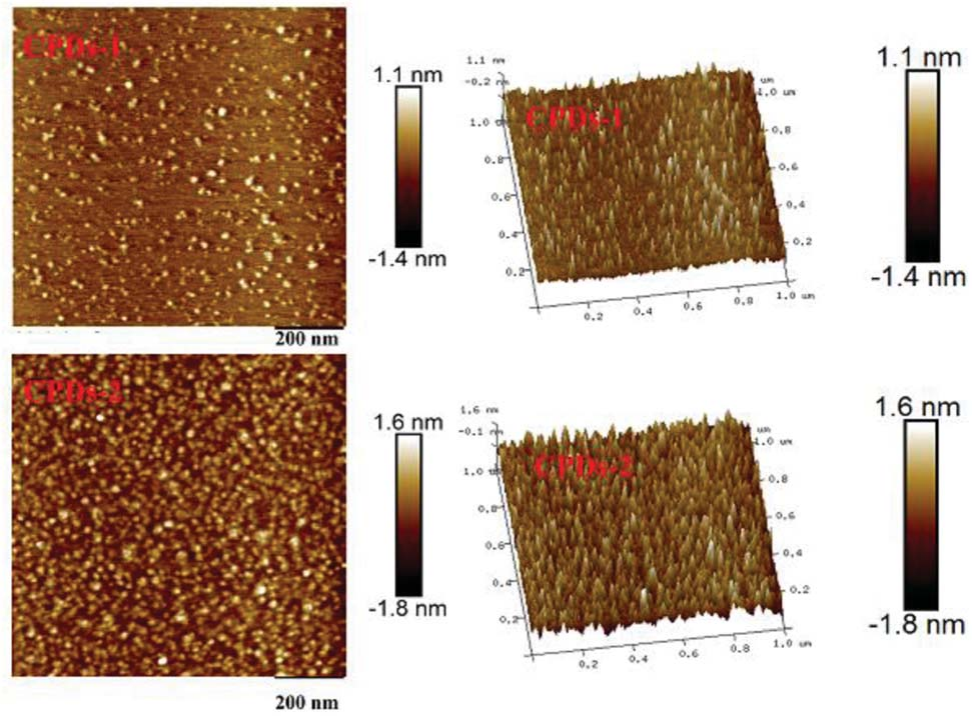
The atomic force microscopy (AFM) images of CPDs-1 and CPDs-2.

Atomic force microscopy (AFM) is a kind of characterization method that can provide a wealth of surface information for materials. AFM images were captured to obtain the morphologies of CPDs-1 and CPDs-2. As shown in Fig. 4, it can be seen that the average height of the monodispersed CPDs-2 is roughly 3.4 nm, which is larger than CPDs-1. Additionally, the granulometric distribution was relatively uniform. The data from AFM supported the above TEM conclusion on the size analysis of the carbonized polymer dots.

### UV-vis spectra of CPDs-1 and CPDs-2

**Fig. 5:**
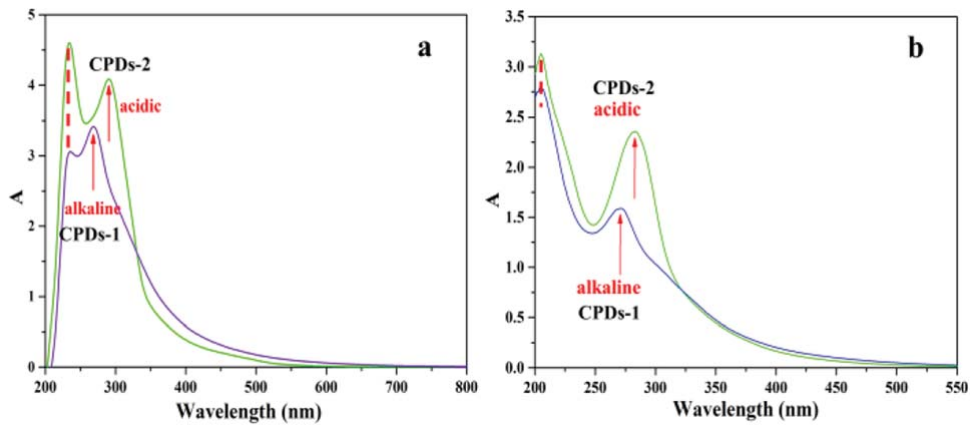
The solid state UV-vis spectra (a) and the liquid UV-vis spectra (b) of CPDs-1 (alkaline condition) and CPDs-2 (acidic condition).

The solid state UV-vis (Fig. 5 a) and liquid UV-vis spectra (Fig. 5 b) of CPDs-1 and CPDs-2 exhibited very similar configuration. The absorption peak at 210 nm was attributed to the π–π* transition from the benzene ring (–C=C–) of the graphitic carbon, and the absorption peak positions in the alkaline and acidic environments were consistent, suggesting that the carbon atom connections inside the particle core under different pH environments were distinctly undifferentiated. However, the UV-vis absorption peak around 260 nm, which can be from the n–π* transition of –C=O, was found to increase to a longer wavelength (300 nm) and represented a significant red shift phenomenon (>20 nm) in the acidic environment. Additionally, the red shift from 260 nm to 285 nm in the liquid UV-vis spectra confirmed the decreasing gaps of the energy levels between the n orbit and π* orbit. We concluded that the absorption peak red shift (n–π*) mainly derived from the surface group changes the CPDs in the acidic and alkaline environments.

### X-ray photoelectron spectroscopy (XPS spectrum), XRD pattern and ^13^CNMR spectrometry

To unveil the chemical bonds and surface groups, the X-ray photoelectron spectroscopy (XPS) spectra of CPDs-1 and CPDs-2 were investigated (Fig. S3†). The detailed high-resolution XPS data were also obtained, which suggested that the nitrogen atom of urea doped into the CPDs. In addition, the peak shapes from the high-resolution XPS of the C, N, and O elements were analogous; however, the hyperfine spectra of the N element showed higher graphitic N and N–O amounts than pyridic N in the acidic environment. The typical XPS characteristic bands in the acidic and alkaline environments indicated that pH value can be an external factor that has a unique influence to the internal structure of CPDs-1 and CPDs-2.

In addition, the XRD diffraction patterns (Fig. S4†) of CPDs-1 and CPDs-2 showed similar broad bands centered around 20°, which were close to the diffraction angle of the graphite plane (002). Moreover, we concluded that the broadness was due to the small diameters of the CPDs. Further, the XRD patterns illustrated that the purity of the sample was also satisfactory.

To discover the surface group differences of CPDs-1 and CPDs-2, the ^13^CNMR (D_2_O) spectra were obtained (Fig. S5†). From the ^13^CNMR spectra of CPDs-1 and CPDs-2, the ^13^CNMR bands from the benzene ring carbon around 130 ppm were discovered clearly, which proved the formation of carbon rings inside the CPDs. In addition, the ^13^CNMR bands at about 190 ppm were attributed to the carbon atoms of –C=O, and the position of –C=O exhibited obvious diversity in the acidic and alkaline environments. Although the ^13^CNMR could not confirm the structures of the CPDs absolutely, it provided some rich information about CPDs-1 and CPDs-2.

### FTIR spectra of CPDs-1 and CPDs-2

**Fig. 6:**
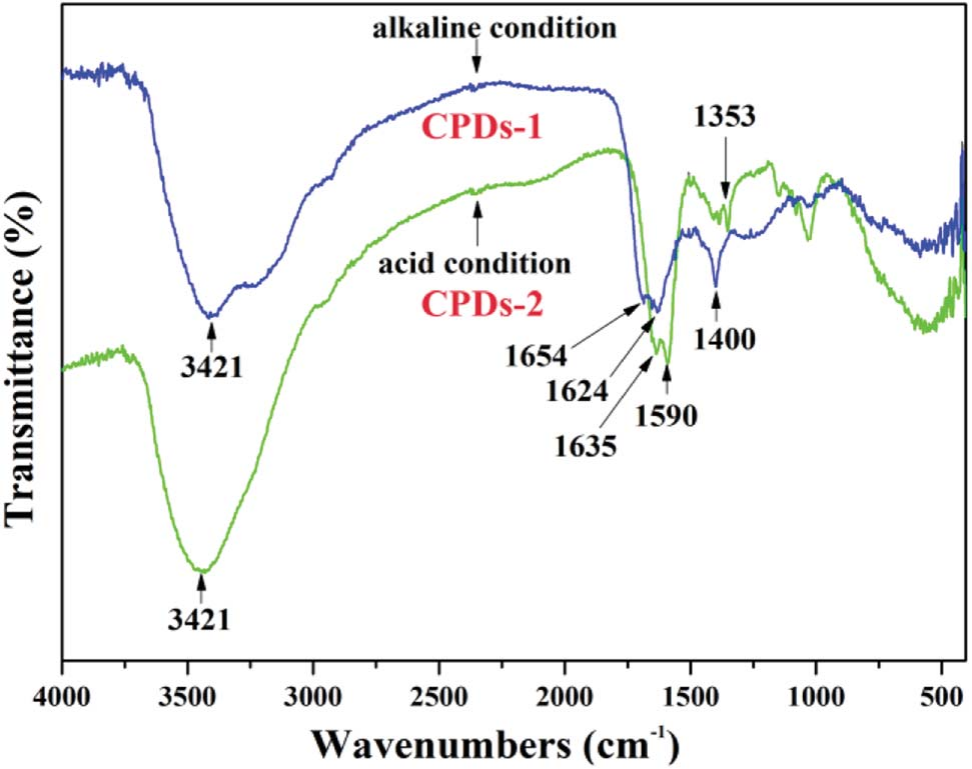
The FTIR spectra of CPDs-1 and CPDs-2.

As shown in the FTIR spectra of CPDs-1 and CPDs-2 from different pH conditions (Fig. 6), the broad absorption peaks at 3421 cm^−1^ are caused by the vibration of –OH, which exists in the surface groups of the nanoparticles. In addition, the IR peak position (3421 cm^−1^) was the same for CPDs-1 and CPDs-2. The strong absorption of CPDs-1 at 1654 cm^−1^ and 1624 cm^−1^ is observed, which was from the stretching vibration groups, however, in the acidic condition, the absorption peaks (1635 cm^−1^ and 1590 cm^−1^) of the C=O and C=N bonds from CPDs showed a significant red shift, indicating that the pH value in the process of the hydrothermal reaction affected the vibration of the –C=O and –C=C– bonds. In addition, the obvious vibrations located at 1353 cm^−1^ and 1400 cm^−1^ corresponded to the vibration of –C–N– bonds, also indicating the large red shift of chemical bonds in different pH environments. FTIR analyses suggested that the vibration variation of the surface chemical bonds of the nanoparticles in different pH value conditions can be an important inducer of bicolor fluorescence activity.

### DFT calculation of the CPDs-1 and CPDs-2 models

**Fig. 7:**
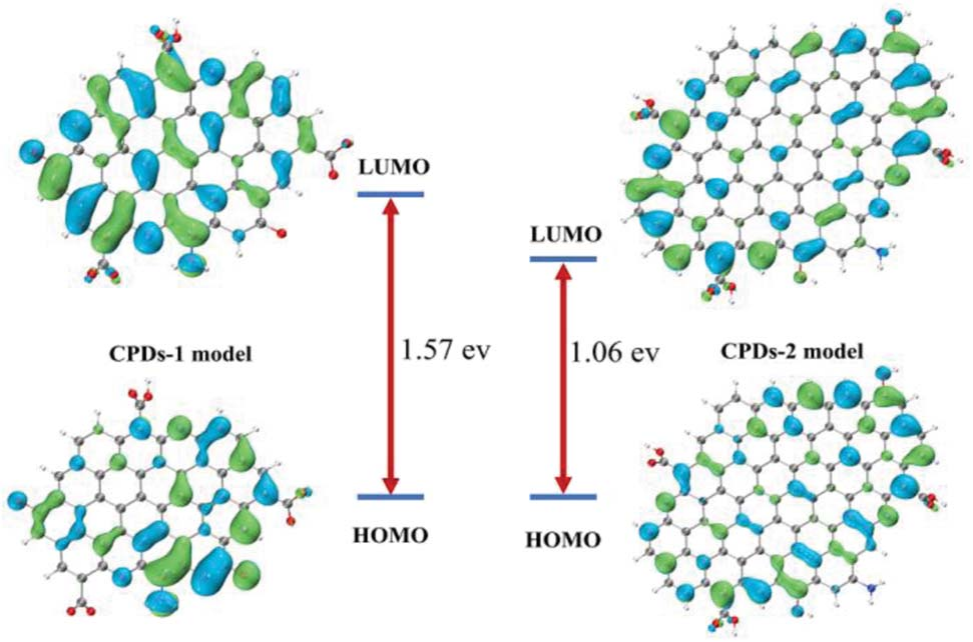
The calculated frontier orbitals for CPDs-1 and CPDs-2, and the HOMO–LUMO gaps of different CPD models from strong acidic and alkaline environments.

**Fig. 8:**
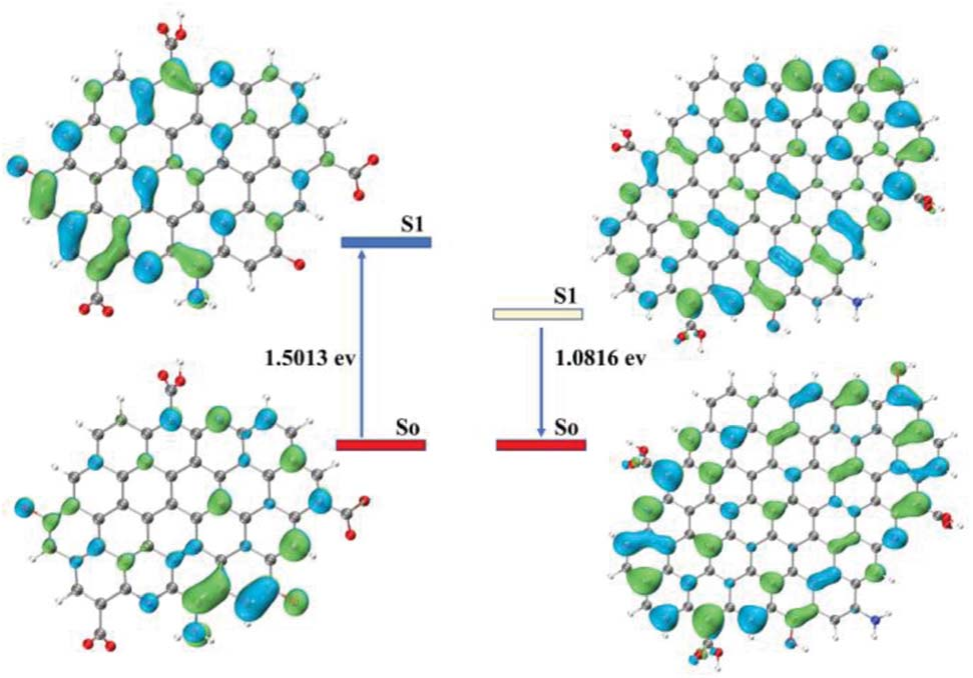
The NTO analysis of S1 orbit transition energy from CPDs-1 (210–211) and CPDs-2 (327–328).

The DFT calculations were performed using Gaussian16 at the PBE0/6-311G(d) level with the D3(BJ) empirical dispersion correction. The TDDFT calculations were carried out with M06-2X/6-311+G(d). The solvent effect of water was examined using the self-consistent reaction field (SCRF) method based on the SMD models.^21^ Based on the core and surface groups of CPDs-1 and CPDs-2, different models have been used to explain the specific structures and activities of the CPDs, with polycyclic aromatic hydrocarbons (PAHs) being the most common CPD model. Lu *et al.* established a series of models to research the relation of structure–activities of the CPDs and gave some satisfactory results.^22^ To explain the interesting bicolor fluorescence behaviour of the CPDs from different pH environments, we also fabricated two CPD models, which were based on the size and surface group differences of CPDs-1 and CPDs-2. According to the DFT calculation, the HOMO–LUMO energy level band gap of CPDs-2 (bigger size) became smaller (1.06 eV), which caused an obvious red shift of the fluorescence emission position (Fig. 7). The DFT calculation results demonstrated that as the size increased, the calculated band gap energy gradually decreased from 1.57 eV to 1.06 eV, which proved the quantum size effect of the CPDs in different pH environments. Such size effects have been observed in many experiments and matched the DFT calculation.^23^ In addition, according to NTO analysis, the single excited state (S1) energy of the two models also exhibited significant differences (Fig. 8). Compared with the S1 orbit energy (1.5013 eV from the 210 orbit to 211 orbit) of CPDs-1, CPDs-2 showed a lower energy state (1.0816 eV from the 327 orbit to 328 orbit), which can induce a longer fluorescence emission wavelength. As such, the results from the DFT calculation demonstrated that strong pH conditions affected the size and surface groups of the CPDs, which caused different fluorescence emission activities. The theoretical models effectively reflected the changing trend of fluorescence emission of CPDs-1 and CPDs-2. However, owing to the complexity of the chemical reaction in the process of CPD formation and the carbon nuclear internal interlayer interaction, it was still a challenging task to develop a more accurate theoretical model to explain the fluorescence activities.

### Cell fluorescence bioimaging of CPDs-1 and CPDs-2

**Fig. 9:**
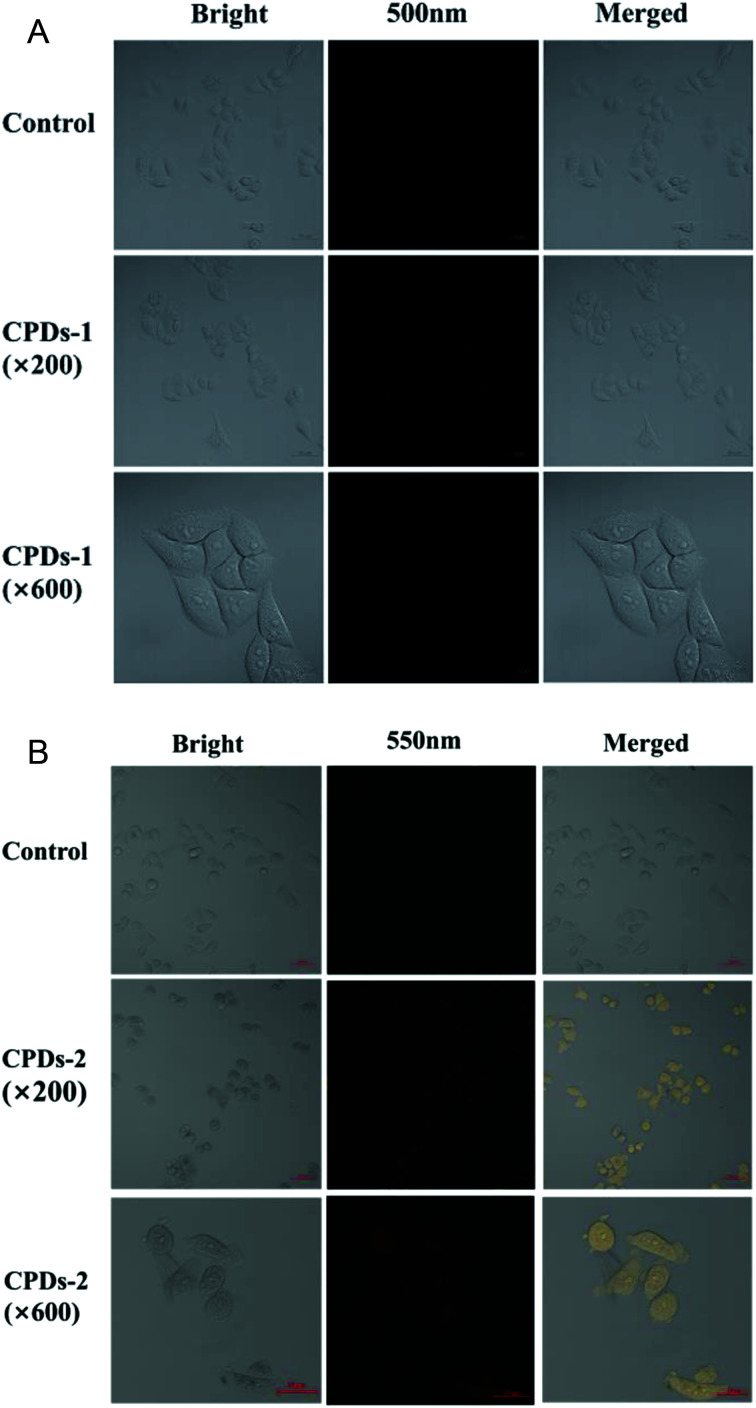
(A) Bright field, blue channel, and overlap confocal fluorescence images of HeLa cells before (control) and after the addition of CPDs-1 (Ex = 405 nm, Em = 500 nm). (B) Bright field, blue channel, and overlap confocal fluorescence images of HeLa cells before (control) and after the addition of CPDs-2 (Ex = 405 nm, Em = 550 nm).

One of the most attractive optical properties of carbonized polymer dots (CPDs) is their bicolor fluorescence activity, which has been applied in biosensors and biomarkers.^24^ To evaluate the fluorescence labelling activities of the CPDs-1 nanoparticles, the cell fluorescence imaging was studied. From the cell fluorescence imaging of CPDs-1 (Fig. 9A), it exhibited a strong blue fluorescence signal, which can permeate the interior of the HeLa cell. Meanwhile, bright yellow fluorescence from the cell imaging of the CPDs-2 nanoparticles was observed (Fig. 9B), which also permeated the cell (×600). The cell morphologies by the fluorescence labelling of CPDs-1 and CPDs-2 were clear and unambiguous. The above cell fluorescence imaging verified their potential application in practical intracellular detection and disease diagnosis.

### Cytotoxicity experiment of CPDs-1 and CPDs-2

To evaluate the cytotoxicity of CPDs-1 and CPDs-2, the cytotoxicity (for HeLa cells) was tested by the CCK8 method (ESI). As shown in Fig. S6,† with the increasing amount (0–80 μL) of CPDs-1 and CPDs-2, CPDs-1 showed relatively lower inhibition rate for HeLa cells compared with CPDs-2. Particularly, in the case of CPDs-1, under the high concentration, the cell inhibition rate is also relatively low, whereas CPDs-2 showed definite toxicity to HeLa cells. This showed that the carbonized polymer dots (CPDs) in different pH environments show differences in cytotoxicity. Therefore, carbonized polymer dots (CPDs) need to be assessed in the process of biological application.

## Conclusions

Herein, we prepared bicolor fluorescence carbonized polymer dots (CPDs) by a hydrothermal method by tuning the pH environment (strongly acidic or alkaline). The bicolor CPDs are from low-cost dextrin and urea. By systematic spectral analysis, we found that the fluorescence emission exhibited consecutive red shift from 480 nm (blue) to 550 nm (yellow). During the nucleation of the carbon nuclei, the particle size and surface groups of the CPDs were closely related to the pH value. Moreover, we concluded that the pH value can affect the nucleation rate of the CPDs. In a strong acidic environment, CPDs showed higher particle size. In strong alkaline conditions, the carbonization and nucleation processes proceeded more slowly, which led to smaller particle sizes. Additionally, we also confirmed that the particles and surface groups caused the fluorescence changes from blue to yellow. Moreover, through constructed models, the fluorescence variation laws were explained, which represented consistency with the calculated orbit energy. In addition, the prepared CPDs showed effective cell bioimaging activities, which proved the potential application of CPDs in organisms. The bicolor fluorescence CPDs provide an ideal platform for imaging and labelling in cells. Besides, the bicolor fluorescence CPDs prepared in strongly acidic and alkaline environments also contribute to useful theoretical guidance in the future.

## Conflicts of interest

There are no conflicts to declare.

## Supplementary Material

RA-012-D1RA08092J-s001
