# Supplementary material for: Preparation, characterization and cell labelling of strong pH-controlled bicolor fluorescence carbonized polymer dots
Source: RSC Adv. 2022 Jan 5;12(3):1258–64. doi: 10.1039/d1ra08092j (PMC8978924; doi:10.1039/d1ra08092j)
Supplement: RA-012-D1RA08092J-s001 [file RA-012-D1RA08092J-s001.pdf]

Electronic Supplementary Material (ESI) for RSC Advances.
This journal is © The Royal Society of Chemistry 2021

Preparation, characterization and cell labelling of strong pH controlled bicolor fluorescence Carbonized Polymer Dots

Zengchen Liu,[a] Like Wang,[a] Baodui Wang,*[b] Yahong Chen,*[a] Fengshou Tian,[a] Yinying Xue,[a] Yanxia Li,[a] Wenping Zhu,[a] Weijie Yang [a]

[a] College of Chemistry and Chemical Eningeering; Henan Key Laboratory of Rare Earth Functional Materials; International Joint Research Laboratory for Biomedical Nanomaterials of Henan; Zhoukou Normal University, Zhoukou 466001, P.R. China, liuzengchen@zknu.cn, Chen-yh75@163.com

[b] State Key Laboratory of Applied Organic Chemistry and Key Laboratory of Nonferrous Metal Chemistry and Resources Utilization of Gansu Province, Lanzhou University, Gansu Lanzhou, 730000, P. R. China. wangbd@lzu.cn

The cytotoxicity (HeLa cells) of CPDs-1 and CPDs-2

The cytotoxicity (HeLa cells) of Cou-In was tested by CCK8 methods. The experiment was divided into control group and experimental group. The control group was added with 100 uL/well complete medium. Added 10 uL, 20 uL, 30 uL, 40 uL, 50 uL, 60 uL, 70 uL, 80 uL CPDs-1 (10 mg/mL) and CPDs-2 (10 mg/mL) to 100 uL/well medium, respectively. Three holes were set for each concentration.

HeLa cells in logarithmic growth phase were taken for cell counting and cell concentration was adjusted. According to the sample, cells were co-cultured with the cells for 24 h. The number of cells in each well was 4×10^3, and the cells were spread into 96-well plates. Cultured overnight in a 37 °C incubator with 5% CO_2. According to the above group processing and training 24 h. Removed the medium. Clean the wells three times with PBS, then add medium containing 10% CCK-8, 5% CO_2 and culture in an incubator at 37°C for 2 hours. The absorbance value at 450 nm was detected by enzyme plate analyzer.

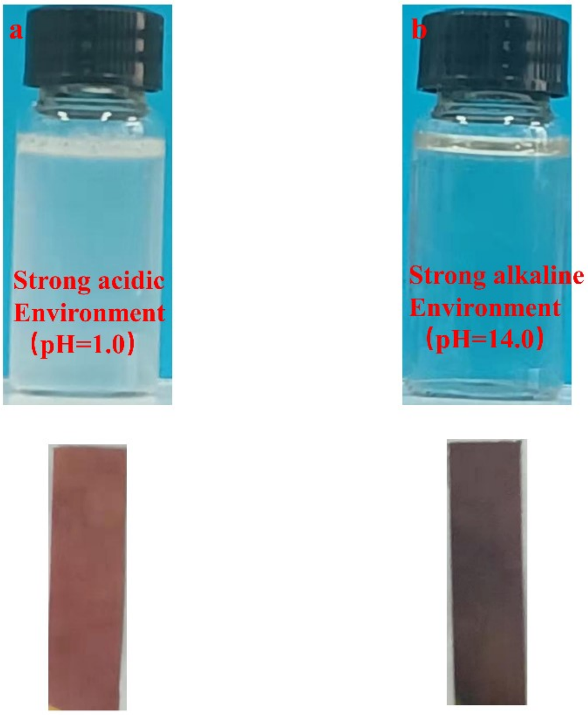

Fig. S1. a. the dextrin and urea water solution in strong acidic environment (pH = 1.0), **b.** the dextrin and urea water solution in strong alkaline environment (pH = 14.0).

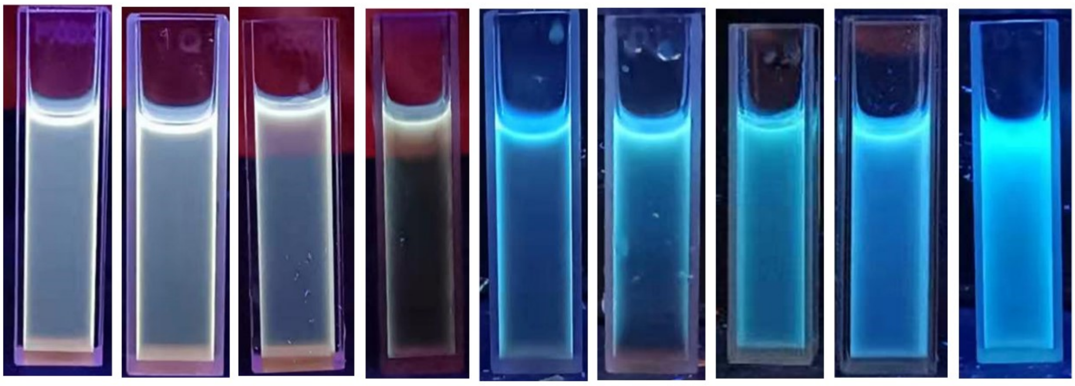

Fig. S2. The fluorescence performances of CPDs in different pH values (pH=14.0, pH=12.0, pH=10.0, pH=8.0, , pH=7.0, pH=6.0, pH=4.0, ,pH=2.0, pH=1.0)

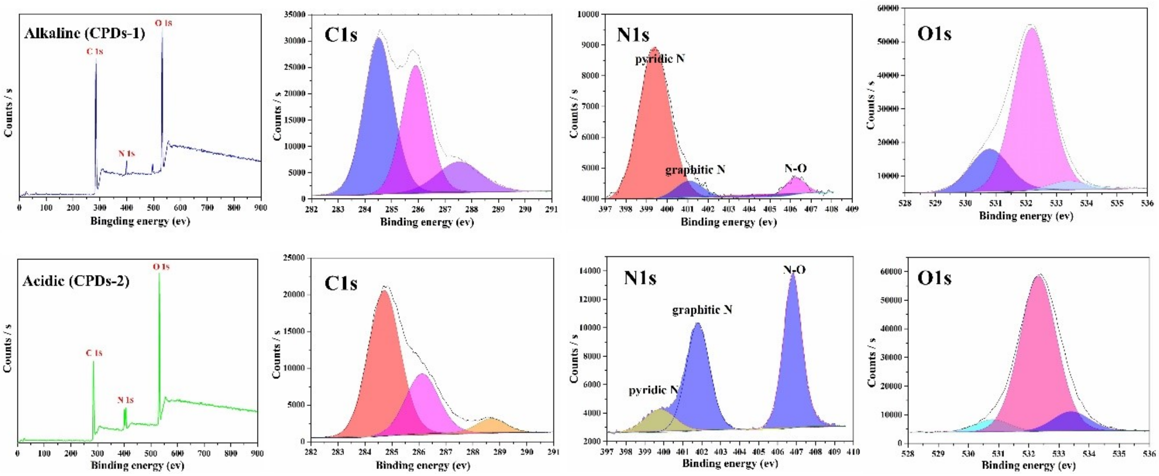

Fig. S3. The XPS spectra of CPDs-1 and CPDs-2, High-resolution XPS spectra of C, N, O elements

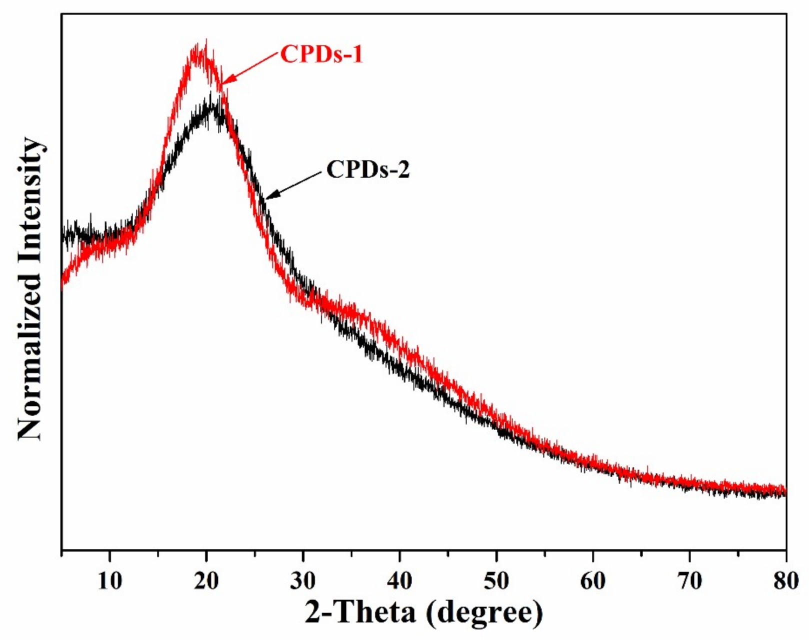

Fig. S4. The XRD patterns of CPDs-1 and CPDs-2

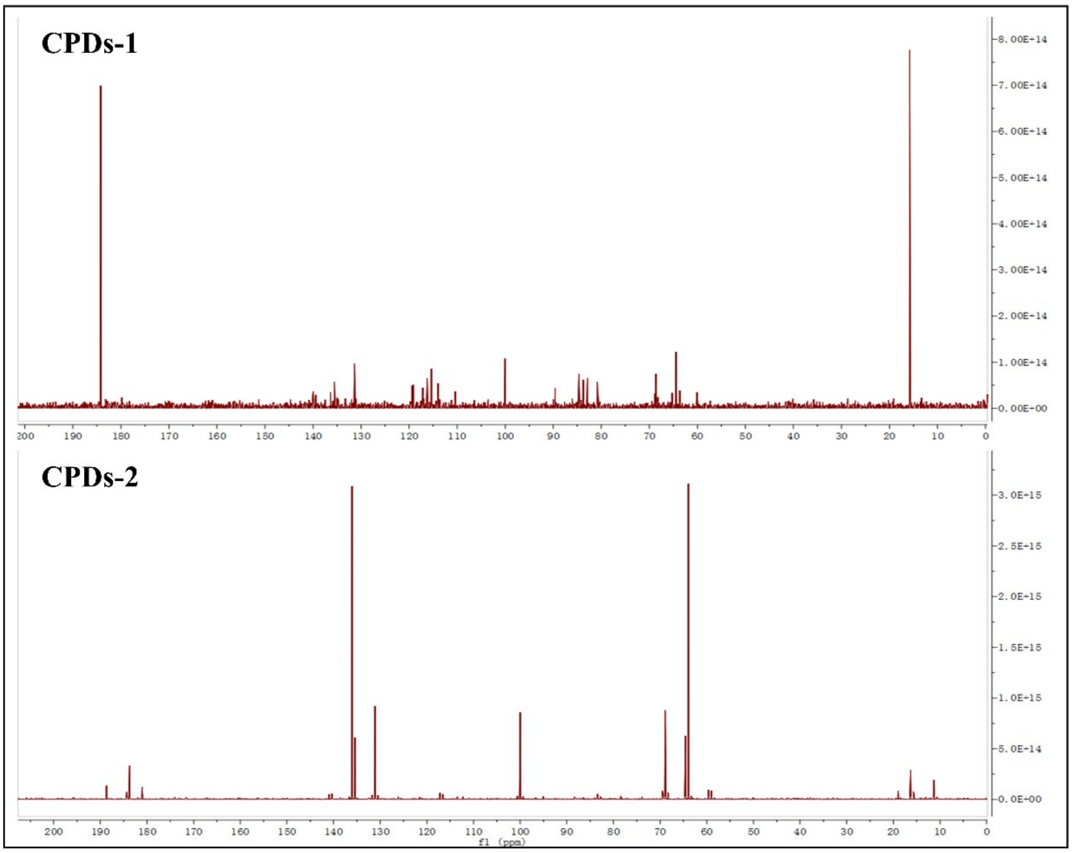

Fig. S5. The ^{13}CNMR (D_2O) patterns of CPDs-1 and CPDs-2

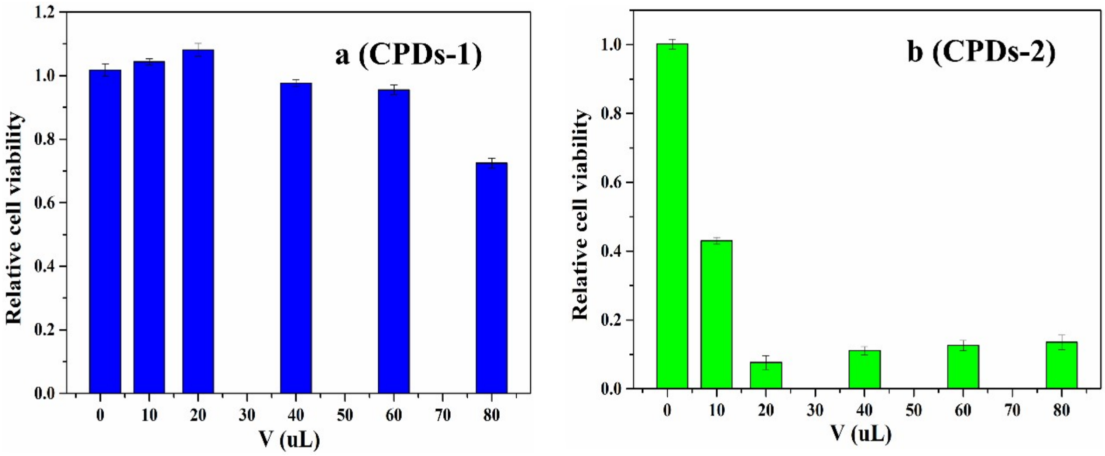

Fig. S6. The cell viability experiments of CPDs-1 and CPDs-2 under different concentrations. The initial concentration of CPDs-1 and CPDs-2 was 10 mg/mL
